# High-Affinity Single-Domain Antibodies for Analyzing Human Apo- and Holo-Transferrin

**DOI:** 10.32607/actanaturae.11663

**Published:** 2022

**Authors:** S. V. Tillib, O. S. Goryainova, A. M. Sachko, T. I. Ivanova

**Affiliations:** Institute of Gene Biology, Russian Academy of Sciences, Moscow, 119334 Russia

**Keywords:** single-domain antibody, nanobody, apo- and holo-transferrin, immunosorbent, affinity chromatography, diagnosis

## Abstract

A highly efficient technology for generating new monoclonal single-domain recombinant antibodies (nanobodies) was used to obtain a panel of nanobodies recognizing human apo- and/or holo-transferrin. This article is devoted to the primary analysis of the properties of two different variants of the new nanobodies obtained by us, as well as to the demonstration of the unique potential of their application for diagnostic studies. The simultaneous use of immunosorbents based on these nanobodies apparently makes it possible to detect changes in the relative abundance of apo- and holo-transferrin in human biological fluids. Such changes could potentially be indicative of an increased risk or degree of development of pathological processes, such as malignant neoplasms in humans.

## INTRODUCTION

Iron is a vital element for a number of key biological processes. Transferrin (Tf) and its receptors (TfR1 and TfR2) are the key proteins regulating iron metabolism in the human body [1]. The high proliferation rate of most tumor cells depends on a supply of sufficient iron and is often associated with increased TfR1 expression [2]. Tf is an 80 kDa glycoprotein composed of two subunits (the N- and C-subunits, 40 kDa each). Each subunit can bind one free ferric ion (Fe^3+^); i.e., up to two iron ions can be attached to Tf. The iron-saturated form of Tf is referred to as holo-Tf. An iron-free form of Tf is known as apo-Tf. The apo-Tf binds Fe^3+^ in blood with high efficiency and transports it to the cell surface for internalization through interaction with TfR [3]. The cell receptor TfR1 binds holo- Tf with high affinity (*K*d1 < 0.1 nM, *K*d2 = 3.8 nM, pH 7.4), while the affinity in case of apo-Tf is ~ 100 times lower (*K*d1 = 49 nM, *K*d2 = 344 nM, pH 7.4) [4]. The complex of iron-bound Tf and receptor formed on the cell surface is internalized by clathrin-mediated endocytosis. The work of the proton pump in the endosomal membrane reduces pH to 5.5 (acidification of the endosome), which triggers conformational changes in both Tf and TfR1, thus leading to the subsequent release of iron from Tf. The ferric iron (Fe^3+^) is converted to ferrous iron (Fe^2+^); the receptor/apo-Tf complex then returns to the cell surface, where apo- Tf is released from its bonding with the receptor at neutral pH [5].

The transferrin iron saturation ratio is a widely used clinical parameter, which is calculated as the ratio between the iron content in the patient’s blood and the indicator of the total iron-binding capacity of serum [6]. It is a rather general characteristic, which does not allow one to capture subtle changes in the relative representation of different forms of transferrin in blood during pathological processes. Specific antibodies may be a more adequate tool for investigating such likely subtle changes. In this work, we describe single-domain antibodies (nanobodies) against various forms of transferrin obtained using a technology developed and used in our laboratory for many years [7, 8, 9, 10].

## EXPERIMENTAL

The peripheral blood plasma of three patients diagnosed with FIGO stage IV ovarian cancer and a urine sample of one patient with invasive bladder cancer were kindly provided by the National Medical Research Center for Radiology of the Ministry of Health of the Russian Federation. Blood plasma from healthy donors was obtained from blood samples taken from employees at a medical laboratory, with their consent, according to the standard protocol. The previously obtained libraries of sequences encoding nanobodies [9] were used in new selection procedures using a modified phage display technique as described previously [7, 8, 9]. Commercial preparations holo-Transferrin human (holo-Tf) and apo-Transferrin human (apo-Tf) procured from Sigma-Aldrich (USA) were used as target antigens. The initially selected sequences of single-domain antibodies were re-cloned and formatted; the nanobodies were then generated in the bacterial periplasm, isolated, and purified. The isolated nanobodies were characterized using electrophoresis and immunoassay (ELISA) [7, 8, 9]. In ELISA, 1-Step Ultra TMB-ELISA reagent (Thermo Scientific, USA) was used for final detection of the secondary HA tag antibodies conjugated to HRP; 2 M sulfuric acid was added, and optical density (OD) was measured at 450 nm. The nanobodies obtained by adaptive re-cloning contain a long linker sequence at the C-terminus (28 amino acid residues of the long variant of the non-canonical camel antibody hinge region), followed by two peptide fragments: a fragment of nine amino acids YPYDVPDYA (HA-tag) and a sequence of six histidine residues (His-tag). The linker linear region contains four conveniently located and easily accessible lysine residues. Using these residues, it is very convenient to carry out chemical reactions to sew other molecules, including the immobilization of a nanobody on BrCN-Sepharose. The nanobodies were cross-linked to CNBr-activated Sepharose 4B (GE Healthcare Life Sciences, USA) according to the manufacturer’s recommendations and as described previously [8, 9]. Hence, new immunosorbents (immunoaffinity columns) were obtained whose specificity depended on the properties of the immobilized nanobody. Immunosorbents with immobilized nanobodies were used to isolate bound proteins as described previously [8, 9].

The binding constants of nanobodies to each form of transferrin (holo-Tf and apo-Tf) in standard phosphate- buffered saline (PBS, pH 7.4) were determined on a MicroCal PEAQ-ITC microcalorimeter (Malvern, Switzerland) using the MicroCal PEAQ-ITC Analysis Software at the Shared Use Center of the Institute of Gene Biology. The fitting model (two sequential binding sites) was unambiguous from the data.

To perform an electrophoretic analysis of the proteins, aliquots of the eluates were collected and analyzed in a 5–19% gradient SDS polyacrylamide gel according to Laemmli. We used the MiniProtean 3 device (Bio-Rad, USA); the power source was Elf-4 (DNA-Technology, Russia). Spectra Multicolor Broad Range Protein Ladder (Thermo Fisher Scientific, USA) was used as a protein marker.

“Native” polyacrylamide gel electrophoresis developed by Novakovsky et al. [11] was adapted for efficient separation of holo-Tf and apo-Tf (in the modification presented on the BraunLab web page – https:// braunlab.de/?page_id=176). The 10% separating and 4% concentrating gels were used. The samples for analysis were prepared by adding 4× loading buffer containing 400 mM Tris-HCl, 600 mM Tris-base, 0.075% Coomasie G-250, 0.025% bromophenol, and 40% glycerol. Only the marker was warmed up before application (in a standard Laemmli SDS buffer). Electrophoresis was carried out at 150 V and cooling (+4°C). At the end of the electrophoresis, the gel was washed in distilled water (three times for 5 min); the proteins were then stained in Imperial™ Protein Stain (Thermo Fisher Scientific).

## RESULTS AND DISCUSSION

**Fig. 1:**
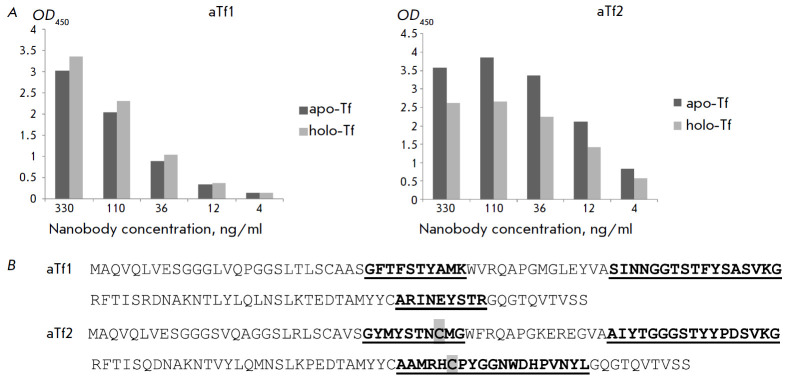
Characteristics of two new anti-human transferrin nanobodies: (A) Immunofluorescent analysis of binding of aTf1 and aTf2 nanobodies in different dilutions to apo-transferrin (apo-Tf, dark bar) or holo-transferrin (holo-Tf, light bar) immobilized in wells of an immunological plate. OD_450_ absorbance values (Y axis) correspond to the number of bound nanobodies and are shown as the average of duplicates with less than 5% variation (the mean background value ~ 0.06 for control wells was subtracted). (B) Amino acid sequences derived from the determined cDNA sequences of the resulting two anti-human transferrin nanobodies. The hypervariable regions of CDR1, CDR2, and CDR3 are underlined. Cysteine residues are detected in the CDR1 and CDR3 regions of the aTf2 nanobody (highlighted in gray)

Using commercially available transferrin preparations, we selected two markedly different major variants of high-affinity nanobodies which had relatively different affinities for holo-Tf and apo-Tf and, apparently, recognized different transferrin epitopes (*Fig. 1, Table*).

**Table:** Determining the binding constants of aTf-1 and aTf-2 nanobodies with two forms of transferrin (holo-Tf and apo-Tf ) in solution at pH 7.4 using a MicroCal PEAQ-ITC microcalorimeter (Malvern)

Nanobody name	Binding to holo-Tf*	Binding to apo-Tf*
aTf1	K_D_1≈ 0.44 nM;	K_D_1≈ 99.4 nM;
	K_D_2 ≈1.44 nM	K_D_2 (not determined)
aTf2	K_D_1≈ 0.94 nM;	K_D_1≈ 0.82 nM;
	K_D_2 ≈ 0.75 nM	K_D_2 ≈ 14.5 nM

^*^Fitting model: sequential binding sites, number of sites – 2.

**Fig. 2:**
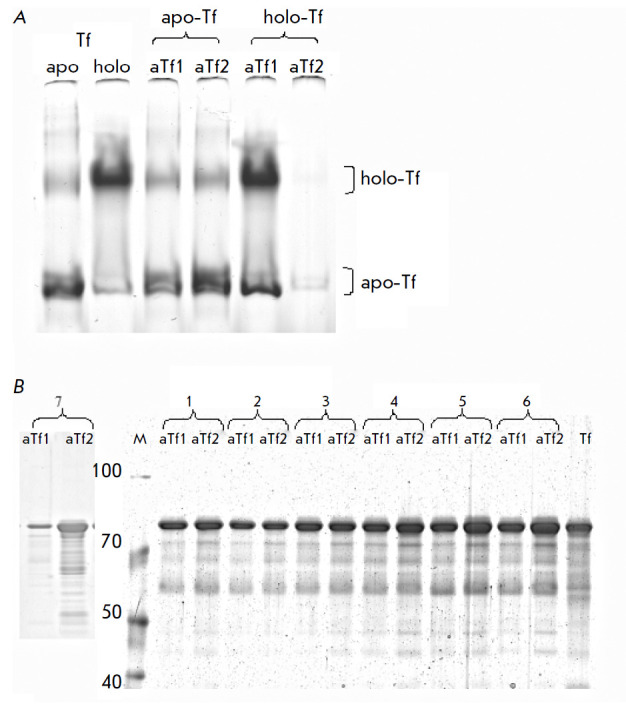
Demonstration of specific binding of different forms of transferrin by nanobodies cross-linked to Sepharose. (A) Gel electrophoresis under conditions preserving the integrity of the transferrin complex with iron ions is used to demonstrate the selective binding of different forms of transferrin (apo-Tf and holo-Tf) to immobilized nanobodies (aTf1 or aTf2). Original transferrins were loaded on two lanes on the left-hand side. (B) Electrophoretic fractionation in 5–19% gradient SDS-polyacrylamide gel of blood (or urine) proteins bound to immunosorbents under physiological conditions and then eluted. Identification of quantitative differences in the representation of transferrin forms apparently differing in iron saturation (all transferrin forms are localized in a given electrophoretic fractionation in one major band) in transferrin fractions isolated simultaneously in parallel using two immunosorbents containing the immobilized nanobodies aTf1 or aTf2 in healthy people (from blood samples denoted by numbers 1, 2, and 3) and cancer patients (with stage 4 ovarian cancer – 4, 5, 6 or in the urine of a patient with muscle-invasive bladder cancer –7). The sizes of the marker bands are indicated in kDa. Tf – transferrin (commercial)

Whereas the difference in binding of different forms of transferrin by the resulting nanobodies was only slightly noticeable in ELISA (*Fig. 1A*), these nanobodies worked unexpectedly selectively in the solution (*Table*) and as a part of an immunosorbent (*Fig. 2A*). The aTf1 nanobody in solution (PBS) at pH 7.4 binds holo-Tf with a very high affinity, while binding apo-Tf is 100 times weaker. Interestingly, the TfR1 receptor interacts with transferrin in a similar manner [4]. Another nanobody, aTf2, binds both forms of transferrin in the solution, but it appears to bind apo- Tf particularly well.

The sequences of these two nanobodies are very different (*Fig. 1B*). In the case of aTf2, CDR3 is significantly increased and there appears to be an additional Cys–Cys bond between CDR1 and CDR3. Both variants of nanobodies were adapted, produced in bacterial periplasm, and purified as described previously [7, 8, 9]. The adapted nanobodies were immobilized on CNBr-sepharose [8, 9], giving rise to two new immunosorbents. The specificities of binding of these immunosorbents to commercial transferrins (apoand holo-) were tested. We adapted and successfully used a special variant of SDS-free polyacrylamide gel electrophoresis to separate iron-bound and noniron- bound transferrins (*Fig. 2A*). To our surprise, in the column format, the aTf2 nanobody barely binds holo-Tf while binding apo-Tf very efficiently. In contrast, the aTf1 nanobody binds holo-Tf very well and binds the purified apo-Tf much weaker than aTf2. Next, immunosorbents in a column format were used in parallel to test potential differences in the relative abundances of the bound forms of transferrin in normal and pathological conditions. The first results of such testing are presented in *Fig. 2*. One can see that while for blood plasma samples from healthy donors (samples 1, 2, and 3) the eluates from both columns contain approximately the same amount of transferrin, for the samples obtained from cancer patients (at advanced stages of ovarian cancer, samples 4–6), one can clearly see that more protein is bound and then eluted in the case of aTf2 nanobodies. A very similar situation is observed when analyzing transferrin in the urine of a patient with an invasive form of bladder cancer (sample 7). In healthy donors, the urinary level of transferrin is ten times lower, and, according to our preliminary observations, we do not see noticeable differences in the amounts of transferrin bound by these two immunosorbents. Hence, this test makes it possible to detect changes in the relative amounts – and availability for binding – of certain epitopes of different transferrin forms using nanobodies. This could probably have a diagnostic potential, including for cancer monitoring; however, the reliability and reproducibility of the proposed test needs to be evaluated on a larger number of samples.

For now, we can only speculate what the observed effects might mean. The immunosorbent with the aTf2 nanobody makes it possible to selectively isolate apo-Tf. Normally, this corresponds to approximately 2/3 of all transferrin subunits contained in blood. The immunosorbent with the aTf1 nanobody preferentially binds holo-Tf and a part (about half) of apo-Tf (this may be Tf with one bound iron ion). As a result, both immunosorbents bind approximately 2/3 of the total plasma transferrin (differing in composition). Cancer cells are known to consume iron particularly efficiently, which can lead to iron deficiency in the biological fluids surrounding the tumor and a relative increase in the proportion of apo-Tf. On the other hand, holo- Tf, unlike apo-Tf, binds very efficiently to the TfR1 receptor on the cell surface. However, TfR1 is also detected in free, extracellular form (as soluble sTfR1 [12]). It cannot be ruled out that in pathological processes such holo-Tf–TfR1 interactions can shield a portion of holo-Tf from binding to the aTf1 nanobody. Taken together, we observe the effect of a prominent increase in the form of transferrin to which the aTf2 nanobody binds but does not bind at all or poorly binds the aTf1 nanobody. We can hypothesize that the exosome-associated increase in apo-Tf in the biological fluid may result from intensive iron consumption by tumor cells.

In conclusion, we note that in this study we have obtained new single-domain antibodies and immunosorbents based on them which differently bind forms of transferrin differing in iron saturation. This ability of differential binding of the resulting immunosorbents makes it possible to observe relative changes in the representation of different transferrin forms that are either directly or indirectly associated with cancer.

## Abbreviations

Holo-Tf and apo-Tf: the iron-containing and iron-free transferrin protein
nanobody: a recombinant protein corresponding to the variable domain of specific camel antibodies, consisting of a homodimer of truncated heavy chains in the absence of immunoglobulin light chains
HA tag: 9 amino acid fragment of YPYDVPDYA to which commercial antibodies are available

## References

[1] Torti S.V., Torti F.M. (2020). Mol Aspects Med..

[2] Candelaria P.V., Leoh L.S., Penichet M.L., Daniels-Wells T.R. (2021). Front. Immunol..

[3] André M.N., Silva A.M.N., Moniz T., de Castro B., Rangel M. (2021). Coordination Chem. Rev..

[4] Kleven M.D., Jue S., Enns C.A. (2018). Biochemistry..

[5] Yiannikourides A., Latunde-Dada G.O. (2019). Medicines (Basel)..

[6] Elsayed M.E., Sharif M.U., Stack A.G. (2016). Adv. Clin. Chem..

[7] Tillib S.V., Ivanova T.I., Vasilev L.A. (2010). Acta Naturae..

[8] Tillib S.V., Privezentseva M.E., Ivanova T.I., Vasilev L.F., Efimov G.A., Gurskiy Ya.G., Georgiev G.P., Goldman I.L., Sadchikova E.R. (2014). J. Chromatogr. B..

[9] Goryainova O.S., Ivanova T.I., Rutovskaya M.V., Tillib S.V. (2017). Mol. Biol..

[10] Tillib S.V. (2020). Mol. Biol..

[11] Nowakowski A.B., Wobig W.J., Petering D.H. (2014). Metallomics..

[12] Speeckaert M.M., Speeckaert R., Delanghe J.R. (2010). Crit. Rev. Clin. Lab. Sci..

